# Relevance of PUFA-derived metabolites in seminal plasma to male infertility

**DOI:** 10.3389/fendo.2023.1138984

**Published:** 2023-05-22

**Authors:** Xiangfeng Chen, Bin Wu, XiaoRong Shen, Xin Wang, Ping Ping, Maohua Miao, Ningning Liang, Huiyong Yin, Huijuan Shi, Jun Qian, Tiancheng Zhang

**Affiliations:** ^1^ Shanghai Human Sperm Bank, Shanghai Key Laboratory for Assisted Reproduction and Reproductive Genetics, Center for Reproductive Medicine, Ren Ji Hospital, School of Medicine, Shanghai Jiao Tong University, Shanghai, China; ^2^ National Health Commission of the PRC (NHC), Key Lab of Reproduction Regulation, Shanghai Institute for Biomedical and Pharmaceutical Technologies, Fudan University, Shanghai, China; ^3^ CAS Key Laboratory of Nutrition, Metabolism, and Food Safety, Shanghai Institute of Nutrition and Health, Chinese Academy of Sciences (CAS), Shanghai, China; ^4^ School of Life Science and Technology, ShanghaiTech University, Shanghai, China; ^5^ Department of Biomedical Sciences, City University of Hong Kong, Hong Kong, China

**Keywords:** male infertility, metabolic profile, normozoospermia, polyunsaturated fatty acids, seminal plasma, sperm

## Abstract

**Aim:**

This study aims to investigate the biological effects of polyunsaturated fatty acid (PUFA)-derived metabolites in seminal plasma on male fertility and to evaluate the potential of PUFA as a biomarker for normozoospermic male infertility.

**Methods:**

From September 2011 to April 2012, We collected semen samples from 564 men aged 18 to 50 years old (mean=32.28 years old)ch., residing in the Sandu County, Guizhou Province, China. The donors included 376 men with normozoospermia (fertile: n=267; infertile: n=109) and 188 men with oligoasthenozoospermia (fertile: n=121; infertile: n=67). The samples thus obtained were then analyzed by liquid chromatography-mass spectrometry (LC-MS) to detect the levels of PUFA-derived metabolites in April 2013. Data were analyzed from December 1, 2020, to May 15, 2022.

**Results:**

Our analysis of propensity score-matched cohorts revealed that the concentrations of 9/26 and 7/26 metabolites differed significantly between fertile and infertile men with normozoospermia and oligoasthenozoospermia, respectively (FDR < 0.05). In men with normozoospermia, higher levels of 7(R)-MaR1 (HR: 0.4 (95% CI [0.24, 0.64]) and 11,12-DHET (0.36 (95% CI [0.21, 0.58]) were significantly associated with a decreased risk of infertility, while higher levels of 17(S)-HDHA (HR: 2.32 (95% CI [1.44, 3.79]), LXA5 (HR: 8.38 (95% CI [4.81, 15.24]), 15d-PGJ2 (HR: 1.71 (95% CI [1.06, 2.76]), and PGJ2 (HR: 2.28 (95% CI [1.42, 3.7]) correlated with an increased risk of infertility. Our ROC model using the differentially expressed metabolites showed the value of the area under the curve to be 0.744.

**Conclusion:**

The PUFA-derived metabolites 7(R)-MaR1, 11,12-DHET, 17(S)-HDHA, LXA5, and PGJ2 might be considered as potential diagnostic biomarkers of infertility in normozoospermic men.

## 1 Background

Semen analysis has moved toward more objective ways of analyzing the composition of the ejaculate, such as sperm numbers, cell morphology, sperm dimensions, sperm function, and the underlying mechanisms that lead to successful fertilization (1). Nevertheless, a routinely performed semen analysis relies on just a few traits, and is a crude method for evaluating male infertility, as seminal composition is affected by environmental factors, infections, and other pathologies. In such cases, the results of semen analysis are either normal or ambiguous.

In approximately 15–30% of male infertility cases, men have demonstrated normal sperm parameters but were still unable to father a child (2). Owing to the increasing need for better tests to evaluate male factor infertility, seminal plasma has received considerable attention, particularly for identifying novel biomarkers (3). Seminal plasma, primarily derived from seminal vesicles, has been considered a valuable repository of a complex set of heterogeneous molecular structures, including proteins, lipids, sugars (fructose), cell-free nucleic acids, ions and small-molecule metabolites, which play a critical role in spermatozoa maturation, nutrition, motility, and function (4, 5). Therefore, analyzing any alterations in seminal plasma composition could aid in additional diagnostic accuracy and an improved understanding of the pathophysiology of male factor infertility (6–8).

There is increasing evidence indicating that the fatty acid composition of seminal plasma might play a role in determining fertility (9). The bulk of literature has shed light on the pivotal role of polyunsaturated fatty acids (PUFAs), including arachidonic acid, linoleic acid, eicosapentaenoic acid ( EPA) and docosahexaenoic acid (DHA) and their metabolites, in sperm biology and spermatogenesis (10–12). Furthermore, the significant effects of PUFA-derived metabolites upon the functional quality of spermatozoa have recently been investigated. A study by Collodel et al. surmised that F_2_ isoprostanes and resolvins represent promising biomarkers for the assessment of semen and follicular fluid quality (13). Nevertheless, semen quality acts as an indirect measure of male fertility, and presently, there is limited research specifically exploring the connection between PUFA metabolites and male fertility outcomes.

Additionally, several studies focusing on oligozoospermic, asthenozoospermic, and oligoasthenoteratozoospermic infertility have performed seminal plasma metabolomics analysis using nuclear magnetic resonance (NMR) (14–16), LC-MS (17), and UHPLC-Q-TOF/MS (18) to identify differential metabolites. However, the relationship between PUFA metabolites and fertility in normozoospermic men is not yet well-established. In this study, we performed LC-MS-based metabolic analysis of 26 PUFA metabolites in seminal plasma, analyzed their relationship with fertility outcomes, and explored their potential as biomarkers for male infertility in normozoospermic men.

## 2 Materials and methods

### 2.1 Study participants

This study employed a random cluster sampling method, selecting 10 towns randomly from the 21 towns in Sandu County, Guizhou Province, from September 2011 to April 2012. The study participants were men aged 18-50(mean=32.28) who were married for the first time and at least one year . Interviewers were trained uniformly by the project team. Using a self-designed questionnaire, the interviewers conducted an in-person interview to collect demographics information. “Fertile” refers to couples who have had at least one offspring in the past. In this study, we defined Infertile as the inability of a couple to achieve a successful pregnancy after one year of regular unprotected sexual intercourse. All participants were asked to abstain from ejaculation for 3–7 days before providing their semen samples. All female partners of male participants underwent ultrasound and gynecological examinations to exclude female infertility factors such as uterine dysplasia.

Data about the age, smoking habits, and BMI of all male participants were obtained during the in-person interviews. After excluding those with left testis and right testis volume of < 12 mL, sperm density < 1 × 10^6^/mL, and abnormal semen agglutination, a total of 564 men were included in this study. This study was approved by the Ethics Committee and the Institutional Review Board(Reference Number: IRB00008297). All the study participants provided written informed consent (**Table S1**).

### 2.2 Semen analysis

Semen samples, collected by masturbation in 25 mL sterile polystyrene jars, were analyzed within 1 h of ejaculation. The semen samples were centrifuged at 3000 rpm for 5 min immediately to primary separate sperm and seminal plasma. Computer-assisted sperm analysis (CASA) (WLJY-9000, Beijing, China) was performed to obtain sperm concentration and motility after the liquefaction of the sample at the clinics, in accordance with the World Health Organization (WHO) guidelines (5^th^ edition) (19). Sperm motility means the total motility (A+B+C). Class A means Rapid progressive motility, where sperm move actively, either linearly or in a large circle, with a velocity of ≥25 micrometers per second. Class B means Slow progressive motility, where sperm move in a less linear or less active pattern, with a velocity of 5-25 micrometers per second. Class C means Non-progressive motility, where sperm move their tails but do not have forward movement or move in a very small circle with a velocity less than 5 micrometers per second. Oligoasthenozoospermia is defined as a condition where the male has less than 15 million sperm per milliliter or 60% of spermatozoa are unable to move forward.

### 2.3 Sample pretreatment and LC-MS analysis

Preliminary separated seminal plasma samples was frozen without preservatives and stored at −20 °C temporarily. All the samples were shipped to the laboratory at the Shanghai Institute of Planned Parenthood Research (Shanghai, China) on dry ice and stored at −80 °C before use. Before LC-MS analysis, we thawed the samples and performed centrifugation at 10,000g for 10 minutes at 22°C. The supernatant was mixed with the internal standard of 1:10 (v/v), and the pH was adjusted to 3.0 using HCl. Equal volumes of acetic acid and amine acetate and 6 mL of the binary mixture, *n*-hexane, and methyl tert-butyl ether were added to a tube and centrifuged. After centrifugation 3000rpm for 8 min at 4°C, the supernatant was transferred to a new tube. A mixture of 6 mL of *n*-hexane and methyl tert-butyl ether was added to the precipitate and centrifuged for another 8 min. The obtained supernatant (organic phase) was combined with the previously obtained supernatant. The combined organic phase was evaporated by drying in a gentle stream of nitrogen. The residue was reconstituted in 50 µL of mobile phase A (water: acetonitrile: formic acid in the ratio of 63:37:0.02, v/v/v) and stored at −80 °C. The fatty acid metabolites were analyzed by LC-MS:Samples were separated on a Phenomenex Kinetix C18 column (3 μm, 100 × 2.1 mm) by using the Thermo Accela UPLC system at a flow rate of 0.4 mL/min with mobile phase A (water: acetonitrile: formic acid in the ratio of 63:37:0.02, v/v/v) and mobile phase B (acetonitrile: isopropanol in the ratio of 50:50, v/v). The gradient was achieved by decreasing the polarity of the mobile phase from 100% to 92% in 6 min and then to 45% within 30 s and was held for 3.5 min. The polarity of the mobile phase A was further decreased to 0% in 3 min and was held for 1 min before returning to 100% in 0.5 min and holding for 1.5 min. Subsequently, mass spectrometry analysis was carried out by using a TSQ Vantage triple-quadrupole mass spectrometer (San Jose, CA, USA). Briefly, an electrospray ionization source was fitted with a stainless-steel capillary (100 μm inner diameter) and the mass spectrometer was operated in a negative-ion mode using multiple reaction monitoring. Data acquisition and analysis were performed using Windows™-based Xcalibur software (Thermo Scientific, version 2.0) (20).

### 2.4 Statistical analysis

Data were analyzed from December 1, 2020, to May 15, 2022. To compare the baseline characteristics of potential confounders between infertile and fertile populations in both oligoasthenospermia and normozoospermia cohorts, including the age, smoking status, sperm concentration, sperm motility, and body mass index (BMI; calculated as weight in kilograms divided by height in meters squared), the chi-square test and *t* test were used for categorical variables and numerical variables, respectively. To increase comparability between populations and decrease the occurrence of false-positive results, we also used propensity score matching (PSM) to obtain an even distribution of baseline characteristics. Propensity score was then calculated by constructing multivariable logistic regression model, in which potential confounders to be adjusted were included as independent variables, and the group assignment (infertile vs fertile) was included as the response variable.

Given that PUFA concentrations are left skewed, it was naturally log-transformed before hypothesis testing and fitting in all statistical models to improve the approximation of the normal distribution. Then, an independent *t* test was performed to analyze differences in the metabolite levels between the oligoasthenospermia infertile vs. oligoasthenospermia fertile, normozoospermia infertile vs. normozoospermia fertile, and oligoasthenotspermia infertile VS normozoospermia fertile groups in the PSM cohort. FDR-adjusted p values below 0.05 (FDR < 0.05) were considered statistically significant.

Based on the above tests, we selected the following potential confounders related to male infertility: age, BMI, current smoking (yes or no), sperm concentration, and sperm motility. We then grouped significantly differential PUFAs in the entire normozoospermia infertile population (without PSM) with median into binary (as an independent variable) and estimated the regression coefficients and 95% confidence intervals (Cis) for normozoospermia infertility risk (whether or not normozoospermic patients were infertile as response variable) using multivariate logistic regression model and with low concentration as the reference. Multivariate regression models were adjusted for the aforementioned confounders.

To investigate the association between PUFAs and sperm concentration or sperm motility, we conducted linear regression models in which sperm concentration or total motility was fitted as the response variable and the PUFA concentration was used as the independent variable.

Before predictor preprocessing, we also performed Spearman’s rank correlation analysis to measure the degree of association between coefficient confounders and PUFAs to reduce collinearity and a potential model overfit. The correlation coefficient of founders above 0.7 was considered as strong correlation, and one of them was removed to fit the logistic regression. Different PUFAs and/or combinations of confounders were used to fit the logistic regression to predict normozoospermia infertility. Then, the performance and comparison of logistic regression models were evaluated using AUC of the ROC curve.

**Figure 1:**
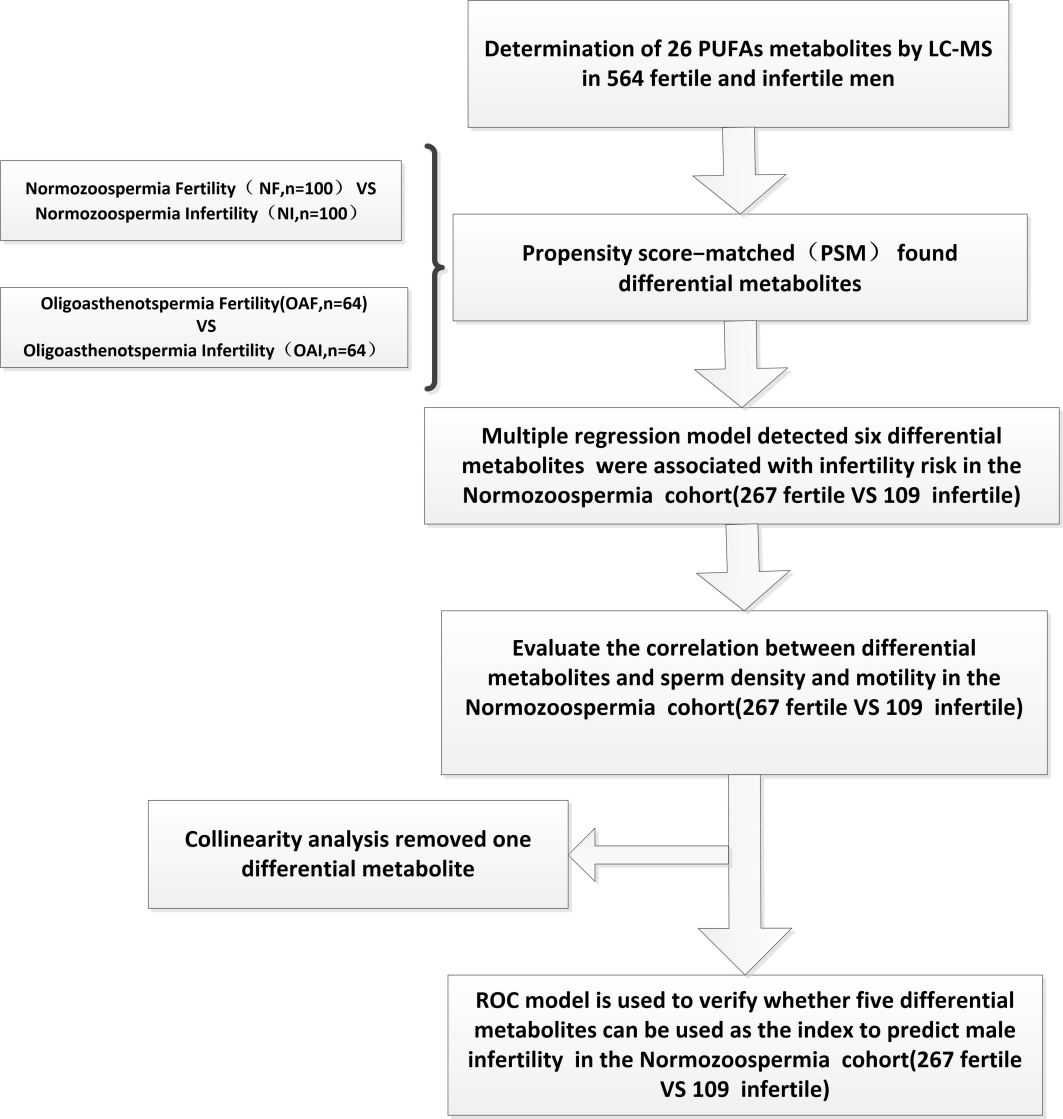
Schematic representation of the stepwise process to the construction of ROC model for predicting male infertility using PUFA-derived metabolites in human seminal plasma.

All analyses were performed using R software version 3.2.3 (R Project for Statistical Computing). All tests were two-sided, and a *P* value of < 0.05 was considered statistically significant. **Figure 1** illustrates the schematic study design.

## 3 Results

### 3.1 Study participants and characteristics of different metabolites

**Table 1** summarizes the demographic and clinical characteristics of study participants. Age, smoking habits, sperm density, sperm motility, and BMI were considered for the analysis. A total of 564 men (N = 564) of 18-50 years old were recruited, including 376 men (n = 376) with normozoospermia, out of which 267 of them were fertile and 109 were infertile, and 188 men with oligoasthenozoospermia, out of which 121 men were fertile and 67 were infertile. The mean ages of fertile and infertile men with normozoospermia were 30.66 ± 6.16 and 34.12 ± 6.81 years, respectively. The mean ages of fertile and infertile men with oligoasthenozoospermia were 32.97 ± 6.13 and 34.52 ± 6.99 years, respectively.

**Table 1:** Demographic and clinical characteristics of study participants.

	Normozoospermia			Oligoasthenotspermia		
	Fertile	Infertile	p	Fertile	Infertile	p
n	267	109		121	67	
Age(years)	30.66 (6.16)	34.12 (6.81)	<0.001	32.97 (6.13)	34.52 (6.99)	0.115
smoke=yes (%)	166 (62.2)	71 (65.1)	0.673	64 (52.9)	41 (61.2)	0.345
Sperm concentration (106 /mL)	66.78 (46.16)	65.23 (39.89)	0.759	28.56 (43.47)	28.87 (33.59)	0.960
Sperm motility(%)	65.89 (14.97)	62.77 (14.64)	0.065	31.02 (17.57)	27.39 (15.65)	0.161
BMI	22.46 (3.14)	22.80 (2.83)	0.322	23.03 (3.40)	22.73 (2.36)	0.530

Values are the mean ± (S.D.).

**Figure 2:**
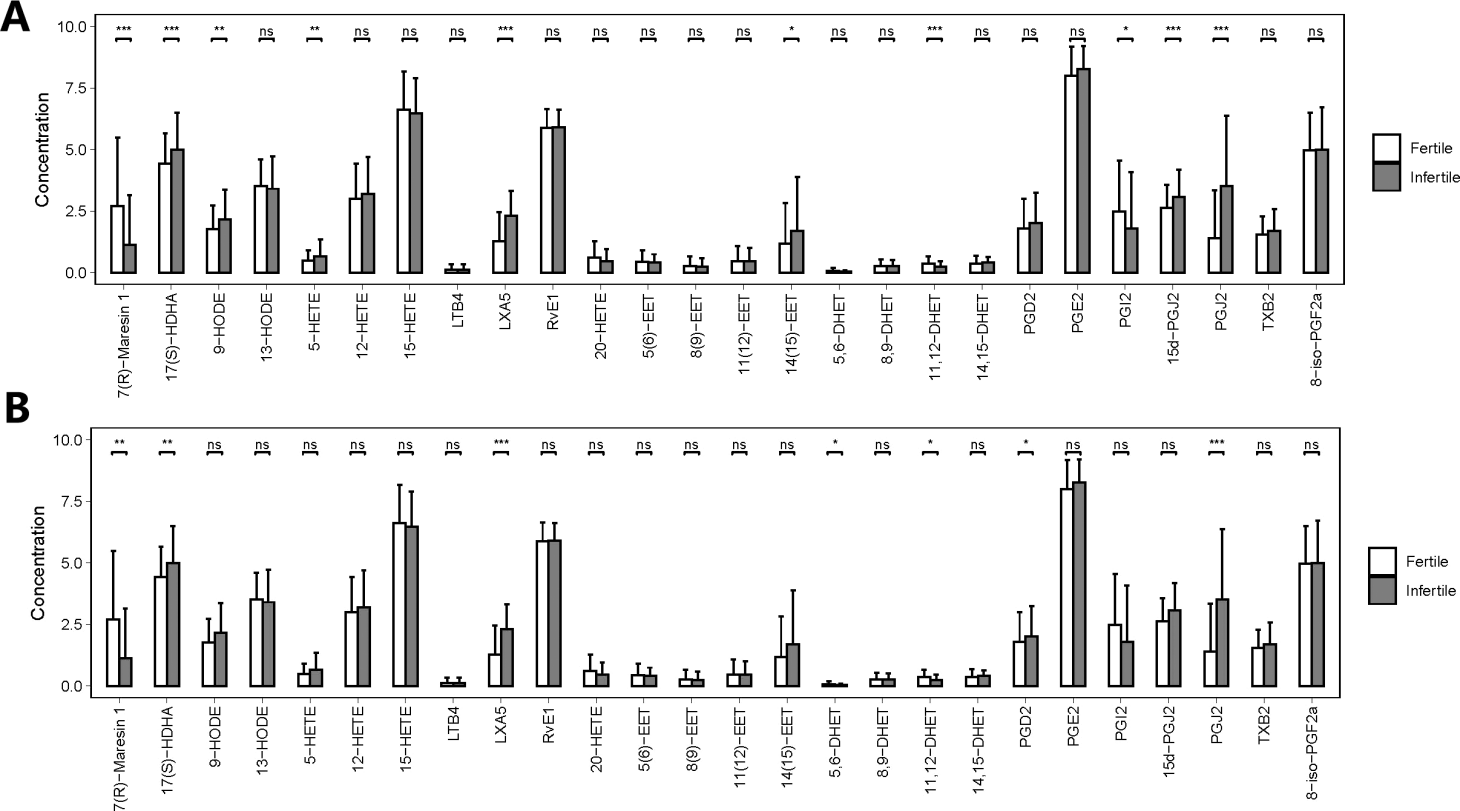
Quantities of PUFA-derived metabolites in seminal plasma of men with different fertility statuses. **(A)** Comparison of PUFA metabolite concentrations in seminal plasma between fertile and infertile men with normozoospermia. **(B)** Comparison of PUFA metabolite concentrations in seminal plasma between fertile and infertile men with oligoasthenozoospermia. The bar charts represent the mean concentrations of each metabolite, with error bars indicating the standard deviation. *, P<0.05; **, P<0.01; ***, P<0.001; ns means P>0.05.

**Figure 3:**
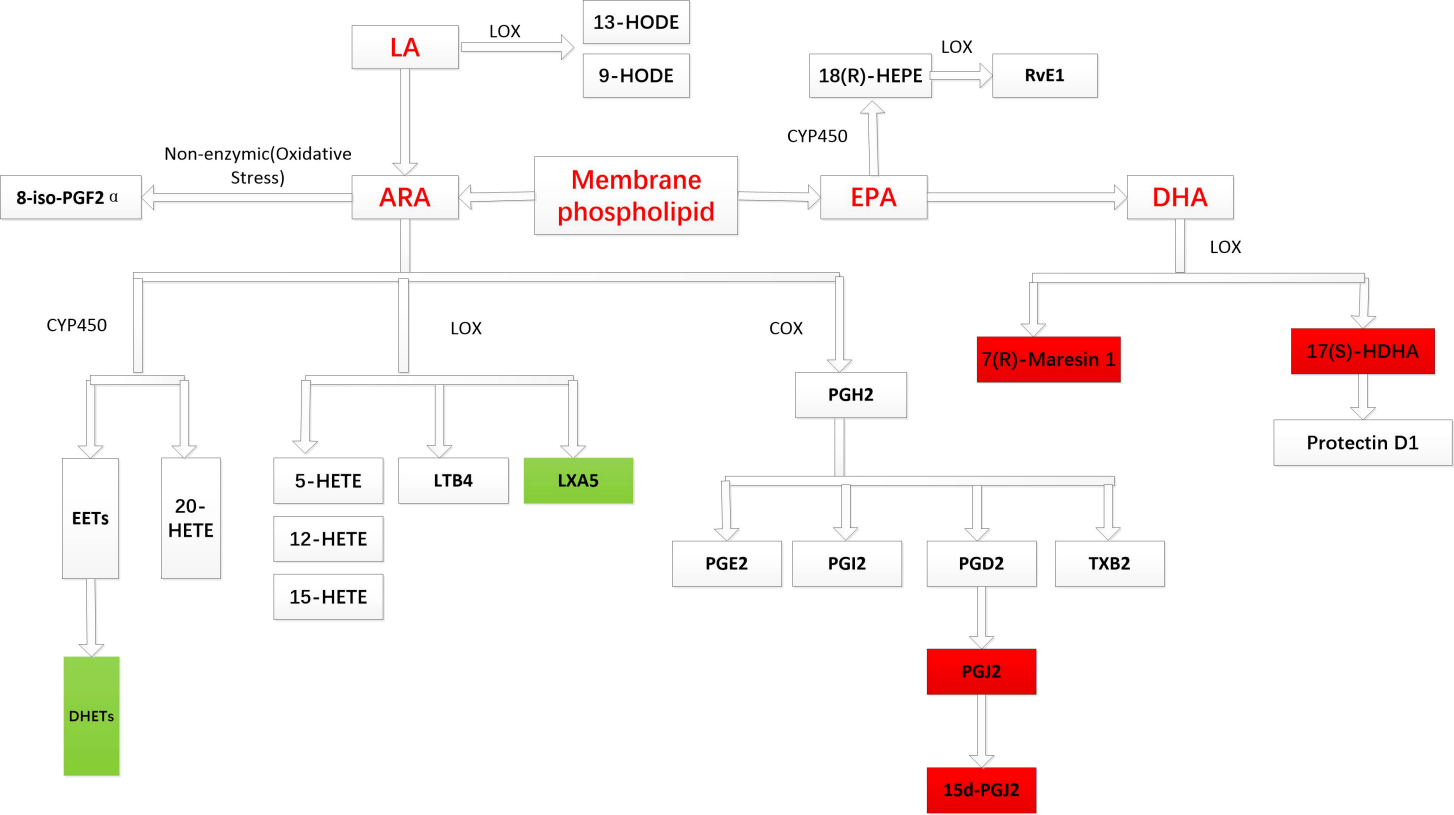
Major metabolic pathways of PUFA-derived metabolites. The red background indicates that an increase in metabolite concentration is associated with an increased risk of male infertility, while the green background indicates that an increase in metabolite concentration is related to a decreased risk of male infertility.

**Figure 2** shows the different PUFA-derived metabolites (N= 26) detected by LC-MS in seminal plasma of fertile and infertile men with normozoospermia and oligoasthenozoospermia. Interestingly, the concentrations of 10 metabolites showed significant differences between fertile and infertile men with normozoospermia, whereas the concentrations of 7 out of 26 metabolites were found to be significantly different between fertile and infertile men with oligoasthenozoospermia. Notably, the concentrations of 10 out of 26 metabolites were found to be significantly different between fertile men with normozoospermia and infertile men with. However, the concentrations of only five metabolites were found to be significantly different among various comparisons between different groups (**Table S2**). The primary synthetic pathways of the 26 PUFA-derived metabolites are shown in **Figure 3**.

### 3.2 Differential metabolic profile of seminal plasma in propensity score-matched cohorts

There were differences in demographic and clinical features at baseline between the fertile and infertile groups. Hence, propensity score matching (PSM) was employed to match groups on covariates to reduce selection bias. After propensity score matching, covariates were balanced (p > 0.05 and standard mean deviation(SMD) value<0.1; **Table 2**) to include a total of 200 normozoospermic men (100 men each in the fertile and infertile groups) and 128 men with oligoasthenozoospermia (64 men each in the fertile and infertile groups). Our analysis of seminal plasma PUFA-derived metabolites from the propensity score-matched cohorts revealed that

**Table 2:** Characteristics of propensity score-matched cohorts.

	Normozoospermia				Oligoasthenotspermia			
	fertile	Infertile	p	SMD	fertile	Infertile	p	SMD
n	100	100			64	64		
Age(years)	33.22 (6.42)	33.16 (6.25)	0.947	0.009	34.45 (5.55)	34.30 (6.85)	<0.001	0.532
Smoke=yes (%)	65 (65.0)	62 (62.0)	0.769	0.062	38 (59.4)	38 (59.4)	0.673	0.062
Sperm concentration (106 /mL)	62.82 (42.58)	66.13 (40.75)	0.576	0.079	31.23 (53.59)	28.58 (34.13)	0.759	0.036
Sperm motility(%)	63.85 (14.73)	63.32 (14.63)	0.801	0.036	28.72 (15.86)	27.79 (15.89)	0.065	0.211
BMI	22.92 (3.10)	22.96 (2.89)	0.916	0.015	22.43 (2.57)	22.75 (2.36)	0.322	0.115

Values are the mean ± (S.D.).

The concentrations of 9 out of 26 metabolites were significantly different between fertile and infertile men with normozoospermia (FDR< 0.05). On the other hand, the concentrations of 7 out of 26 metabolites were significantly different between fertile and infertile men with oligoasthenozoospermia (FDR < 0.05). However, the statistical significance of three metabolites, 5-hydroxyeicosatetraenoic acid (5-HETE), 20-hydroxy-5, 8, 11, 14-eicosatetraenoic acid (20-HETE), and prostaglandin I2 (PGI2) was found to be different before and after matching, which indicated that the differential expressions of these three metabolites in seminal plasma of men with normozoospermia might be related to the presence of different confounding factors.

**Figure 4:**
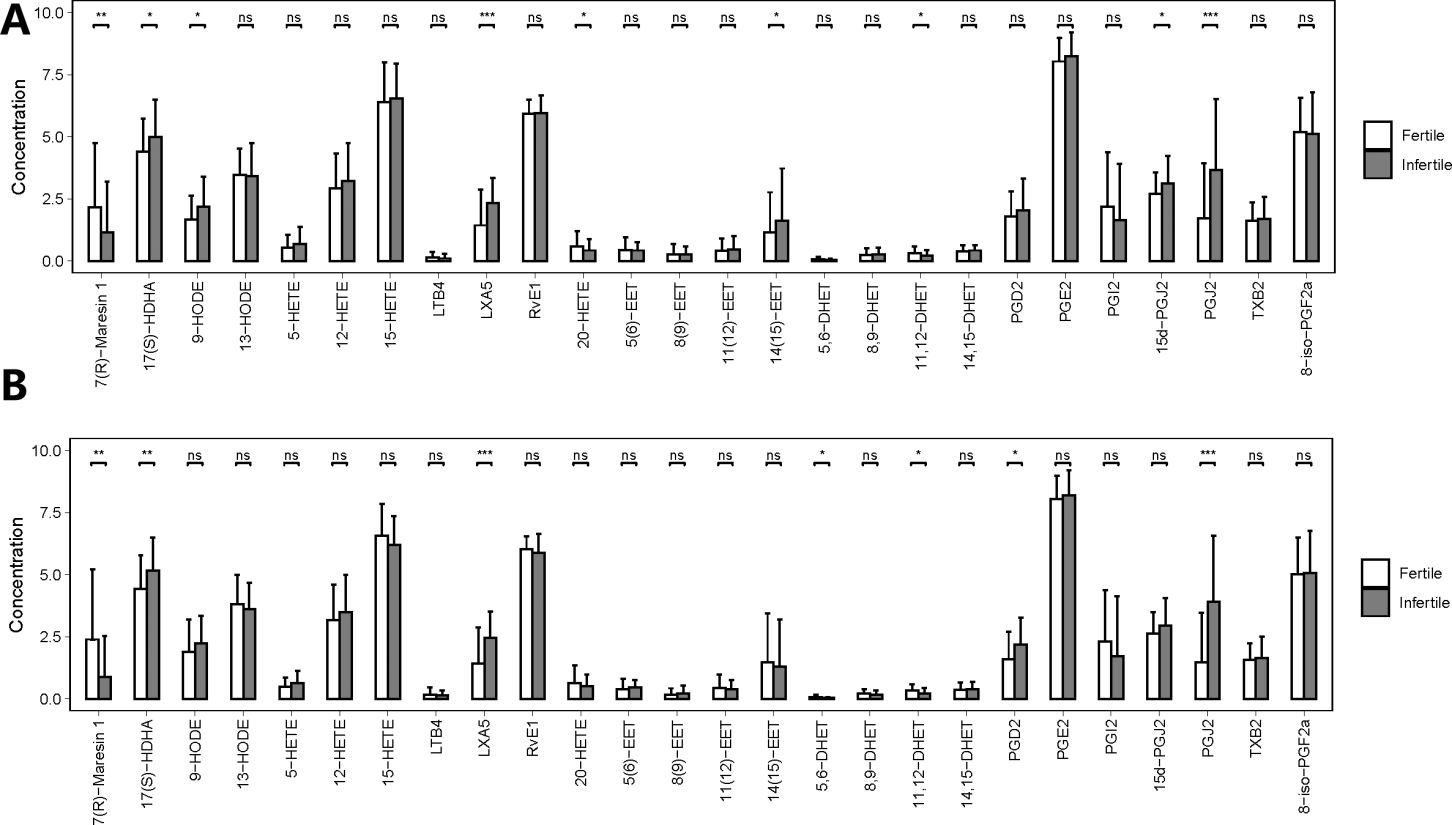
Quantities of PUFA-derived metabolites in seminal plasma of men with different fertility statuses in propensity score-matched cohorts. **(A)** Comparison of metabolite concentrations in seminal plasma between fertile and infertile men with normozoospermia in matched cohorts. **(B)** Comparison of metabolite concentrations in seminal plasma between fertile and infertile men with oligoasthenozoospermia in matched cohorts. The bar charts represent the mean concentrations of each metabolite, with error bars indicating the standard deviation. *, P<0.05; **, P<0.01; ***, P<0.001; ns means P>0.05.

We also noticed that the concentrations of 15d-PGJ2, 14,15-epoxy-5Z,8Z,11Z-eicosatrienoic acid (14 (15)-EET), 20-HETE and 9-hydroxyoctadecadienoic acid (9-HODE) (Label with only b) showed statistically significant differences only between fertile and infertile men with normozoospermia, the concentrations of 5,6-dihydroxy-8Z,11Z,14Z-icosatrienoic acid (5,6-DHET), and prostaglandin D2 (PGD2) (**Figure 4**, **Table S3**) were found to be significantly different only between fertile and infertile men with oligoasthenozoospermia. Further, propensity score matching for comparing the seminal plasma metabolic profile of fertile men with normozoospermia with that of infertile men with oligoasthenozoospermia could not be performed owing to the presence of confounding factors, like sperm concentration and motility, which may give rise to false-positive results, with a differential metabolic profile.

### 3.3 Examining the associations of seminal plasma metabolites’ levels for predicting the risk of infertility in men with normozoospermia

**Figure 5:**
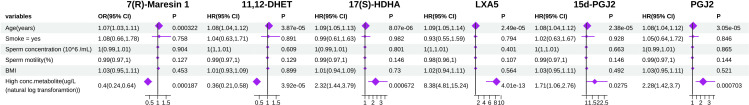
Forest plot showing adjusted coefficient or Odds ratio (HR) and 95% confidence interval (CI) for PUFA-derived metabolites between highest 50% and lowest 50% normozoospermia.

Next, we focused on determining the associations between the concentrations of seminal plasma metabolites and the risk of infertility in men with normozoospermia. In a logistic regression model adjusted for potential confounding factors, such as age, smoking history, sperm density, sperm motility, and BMI, the concentrations of six seminal plasma metabolites were significantly associated with the risk of infertility in a total of 376 men with normozoospermia. We found that, in men with normozoospermia, higher concentrations of 7(R)-MaR1 and 11,12-DHET were significantly associated with a reduced risk of infertility compared with that of those with lower concentrations of these metabolites (odds ratio (HR): 0.4 (95% CI [0.24,0.64]) and HR: 0.36 (95% CI [0.21,0.58]), respectively. On the contrary, increased concentrations of the other four metabolites, 17(S)-HDHA (HR: 2.32 (95% CI [1.44,3.79]), LXA5 (HR: 8.38 (95% CI [4.81,15.24]), 15d-PGJ2 (HR: 1.71 (95% CI [1.06,2.76]), and PGJ2 (HR: 2.28 (95% CI [1.42,3.7]) were found to be significantly associated with an increased risk of infertility in men with normozoospermia. In addition, we observed that age was a strong confounding factor that significantly influenced the association of metabolite concentration with the risk of male infertility (**Figure 5**).

### 3.4 Validating the six metabolites as potential biomarkers of infertility in men with normozoospermia

Further, we investigated the possible correlations between the levels of PUFA-derived metabolites in seminal plasma and different semen parameters. Interestingly, we found that there were statistically significant linear relationships between the concentrations of a few metabolites and sperm density (13-HODE, 14,15-DHET, PGD2, PGE2, 15d-PGJ2, TXB2, 8-iso-PGF2α), sperm motility (9-HODE, 13-HODE, 5-HETE), and both sperm density and motility (13-HODE) among men with normozoospermia. However, among the six differential metabolites observed in men with normozoospermia, a statistically significant linear relationship was observed between the levels of 15d-PGJ2 and sperm density (**Table 3**). On the contrary, no significant linear correlations were observed between the levels of the other five metabolites and sperm density and/or sperm motility.

**Table 3:** The linear relationship between PUFA-derived metabolites and sperm density and motility in men with normozoospermia.

	sperm density		sperm motility		
	coefficent	P value	coefficent	P value	
LOX PATHWAY MATABOLITES					
7(R)-Maresin 1	0.22	0.80	-0.07	0.81	
17(S)-HDHA	-0.24	0.89	-0.21	0.71	
9-HODE	-4.06	0.061	-1.61	0.028	
13-HODE	-5.13	0.0092	-1.42	0.034	
5-HETE	-2.67	0.54	-3.41	0.024	
12-HETE	-0.16	0.92	-0.53	0.32	
15-HETE	1.54	0.31	0.42	0.41	
LTB4	-5.99	0.58	0.76	0.83	
LXA5	-2.97	0.11	-0.49	0.43	
RvE1	-5.11	0.096	-0.16	0.88	
P450 PATHWAY MATABOLITES					
20-HETE	-3.21	0.38	0.20	0.87	
5(6)-EET	7.08	0.17	2.06	0.24	
8(9)-EET	4.71	0.44	1.24	0.54	
11(12)-EET	3.97	0.31	0.55	0.67	
14(15)-EET	0.36	0.77	0.30	0.48	
5,6-DHET	20.34	0.34	-3.63	0.61	
8,9-DHET	11.63	0.18	1.86	0.52	
11,12-DHET	7.96	0.32	0.96	0.72	
14,15-DHET	21.48	0.0076	-2.09	0.44	
COX PATHWAY MATABOLITES					
PGD2	-7.23	9.8E-05	-0.69	0.28	
PGE2	-5.54	0.0070	0.45	0.52	
PGI2	0.38	0.72	0.33	0.36	
15d- PGJ2	-8.25	0.000303	-0.69	0.37	
PGJ2	-1.33	0.16	-0.33	0.30	
TXB2	-11.96	2.9E-05	-0.31	0.75	
Non-enzymatic MATABOLITES					
8-iso-PGF2α	-3.62	0.01	-0.22	0.65	

Next, we performed Spearman’s rank correlation analysis to measure the degree of association between two variables and reduce collinearity. Our results showed a high degree of collinearity between the levels of PGJ2 and 15d-PGJ2 (**Supplementary Figure 1**). Therefore, PGJ2 was kept in the following analysis.

**Figure 6:**
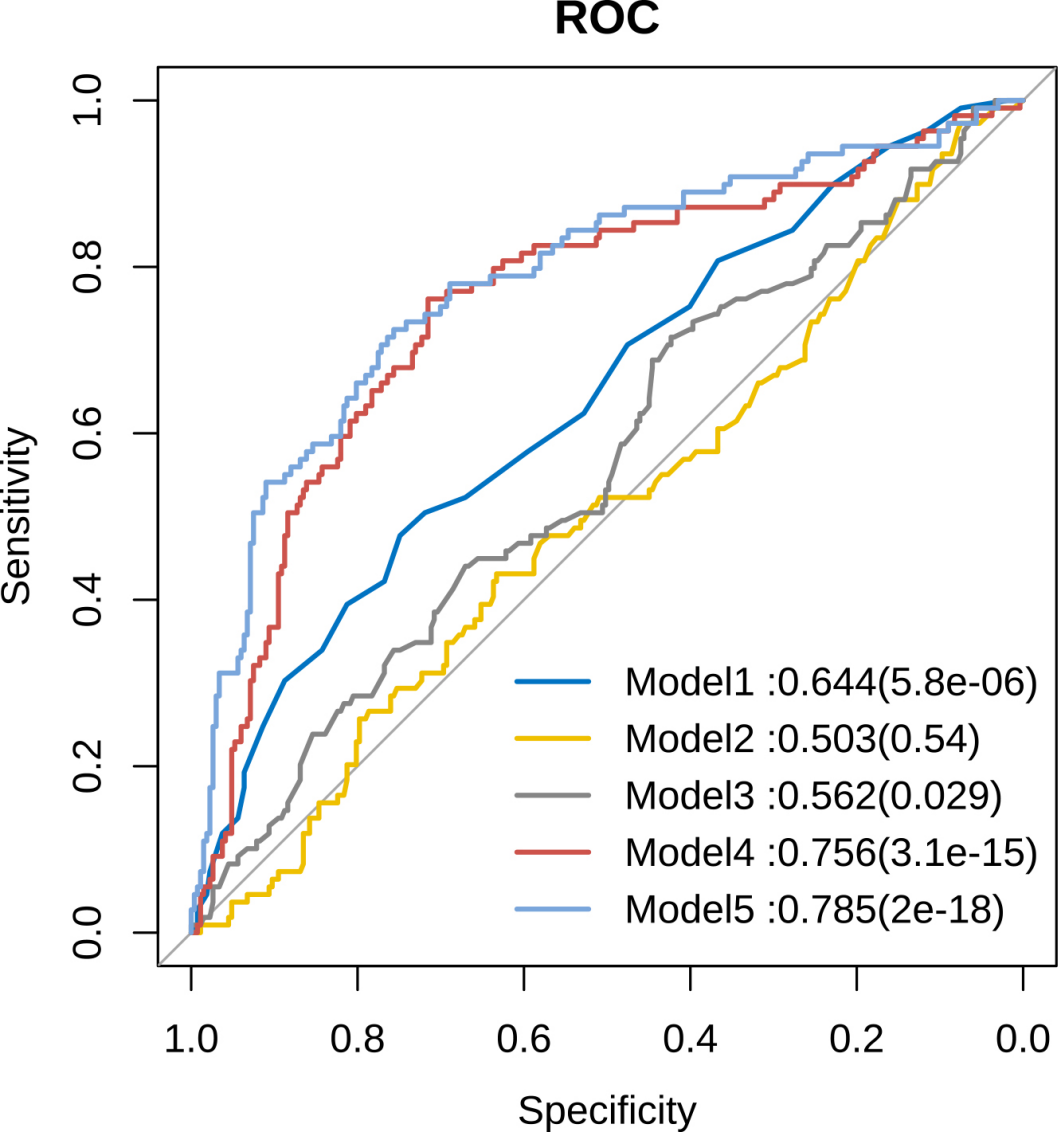
Comparison of sensitivity and specificity for infertility prediction by PUFA-derived metabolites. Model 1: Age, Model 2: Sperm concentration, Model 3: Sperm motility, Model 4: Five differential metabolites, Model 5: Five differential metabolites + age + sperm concentration + sperm motility.

Furthermore, we employed receiver operating characteristic (ROC) curves to determine the diagnostic accuracy of metabolites in predicting the risk of male infertility (**Figure 6**). We found that the values of area under the curve (AUC) were less than 0.7 for models 1–3 considering age, sperm density, and sperm motility, respectively, whereas the value of AUC for model 4 (constructed with five metabolites :7(R)-Maresin 1,11,12-DHET, 17(S)-HDHA, LXA5, and PGJ2) was 0.756. However, the value of AUC for model 5 with a combination of PUFA metabolites, age, sperm density, and sperm motility was 0.785 (**Figure 6**). The performance of this model was found to be slightly improved though not substantial.

## 4 Discussion

In this study, we employed LC-MS, an ultra-highly sensitive technique with a broad dynamic range that requires low metabolite concentrations in samples, to investigate the differences in seminal metabolomics patterns of 26 bioactive PUFA-derived metabolites between fertile and infertile men with normozoospermia and oligoasthenozoospermia. Most importantly, we identified six differential PUFA-derived metabolites namely, 7(R)-MaR1, 11,12-DHET, 17(S)-HDHA, LXA5, 15d-PGJ2, and PGJ2 in a balanced propensity score-matched cohort. The levels of these metabolites were not only significantly different between fertile and infertile men with normozoospermia but also were significantly associated with the risk of male infertility in the unbalanced whole population.

Our study demonstrated that the concentrations of 15d-PGJ2, 14 (15)-EET, 20-HETE, and 9-HODE were found to be significantly different only between fertile and infertile men with normozoospermia; the concentrations of 5,6-DHET and PGD2 were significantly different only between fertile and infertile men with oligoasthenozoospermia. Our results are consistent with that of a previous study by Lazzarino et al., which showed that 21/26 low molecular weight compounds assayed in seminal plasma of infertile males were significantly different from corresponding values in fertile controls (21). Collectively, our results suggest that the metabolic profile of seminal plasma gets altered when different anomalies exist in the spermiogram.

SPMs (specialized pro-resolving lipid mediators) are enzymatically biosynthesized by oxygenating PUFAs and include four families: lipoxins, resolvins, protectins, and maresins (22–24). Previous studies have shown that the concentration of SPMs increases in the seminal plasma of infertile men, accompanied by a decrease in sperm quality (25, 26). In this study, we identified that the concentrations of 2 SPMs, namely LXA5 of the lipoxin family and 7(R)-MaR1 of the maresins family, were significantly different between infertile and fertile men with normozoospermia and were also significantly associated with the risk of infertility. In addition, 17(S)-HDHA, a precursor of protectins, was also identified as risk factors for infertility. However, resolvins E1(RvE1) were not found to be significantly associated with the risk of infertility in normozoospermic men.

In this study, we did not observe a significant linear correlations between the levels of most differential metabolites and sperm density and/or sperm motility. These results suggest that assessment of seminal plasma metabolic status may be independently performed in addition to routine semen analysis for detecting infertility in normozoospermia. Furthermore, we did not observe any significant correlations between the concentrations of LXA5 or 7(R)-Mar1 and sperm density and/or motility, suggesting that SPMs may affect the reproductive outcome through other mechanisms without influencing sperm quality. It is well-known that the sperm-uterine interaction affects the reproductive outcome (27). Upon contact with the female tissues, seminal fluid elicits a controlled inflammatory response that affects several aspects of reproductive function to ultimately maximize the chances for the male factor to produce healthy offspring (27). As SPMs act as key inflammatory regulators (28, 29), we speculated that abnormal levels of SPMs in seminal plasma may lead to an adverse response in the female tissue, thus negatively impacting male fertility.

Further, we determined the diagnostic accuracy of the 5 differential metabolites (7(R)-maresin 1, 11,12-DHET,17(S)-HDHA, LXA5 and PGJ2) in predicting the risk of male infertility using ROC curves. Notably, the value of AUC for the model constructed using PUFA-derived metabolites, excluding age, sperm density, and sperm motility, was 0.756. This finding suggests that the predictive accuracy of the 5 differential metabolites might be better compared to that of semen analysis (AUC=0.503 or 0.562) in infertile men with normozoospermia.

## 5 Limitations

Although our study carried out routine ultrasound and gynecological examinations for male spouses, it could not exclude the influence of female factors caused by other factors on this study. Therefore, one major limitation of this study is that the influence of female factors on reproductive outcome was not fully considered. So, the results of this study should be interpreted carefully. This could also explain why the AUC values in our ROC models did not exceed 0.9.

## 6 Conclusions

In this study, using an LC-MS-based metabolomic analysis platform, we explored the biological association between PUFA-derived metabolites in seminal plasma and male infertility. Our findings also provide preliminary evidence supporting the potential diagnostic utility of measuring PUFA-derived metabolites in seminal plasma to predict infertility in normozoospermic men and further research is needed to confirm these findings.

## Data availability statement

The raw data supporting the conclusions of this article will be made available by the authors, without undue reservation.

## Ethics statement

The studies involving human participants were reviewed and approved by Ethics Committee and Institutional Review Board of Shanghai Institute of Planned Parenthood Research. The patients/participants provided their written informed consent to participate in this study.

## Author contributions

XC: data analysis, data interpretation, manuscript preparation. BW: data collection. XS: data collection. XW: raw data processing. PP: raw data processing. MM: revised the manuscript for intellectual content. NL: sample pretreatment. HY: revised the manuscript for intellectual content. HS: data interpretation, revised the manuscript for intellectual content. JQ, TZ: study design, data interpretation, revised the manuscript for intellectual content.

## Supplementary material

Supplementary Figure 1 Analysis of collinearity among different risk factors.

Supplementary Table 1 Raw data used in the study.

Supplementary Table 2 Values of PUFA-derived metabolites determined by LC-MS in the seminal plasma of men with normozoospermia.

Supplementary Table 3 Values of PUFA-derived metabolites in propensity score-matched cohorts.
