# Supplementary figures and images for: Relevance of PUFA-derived metabolites in seminal plasma to male infertility
Source: Front Endocrinol (Lausanne). 2023 May 22;14:1138984. doi: 10.3389/fendo.2023.1138984 (PMC10240070; doi:10.3389/fendo.2023.1138984)

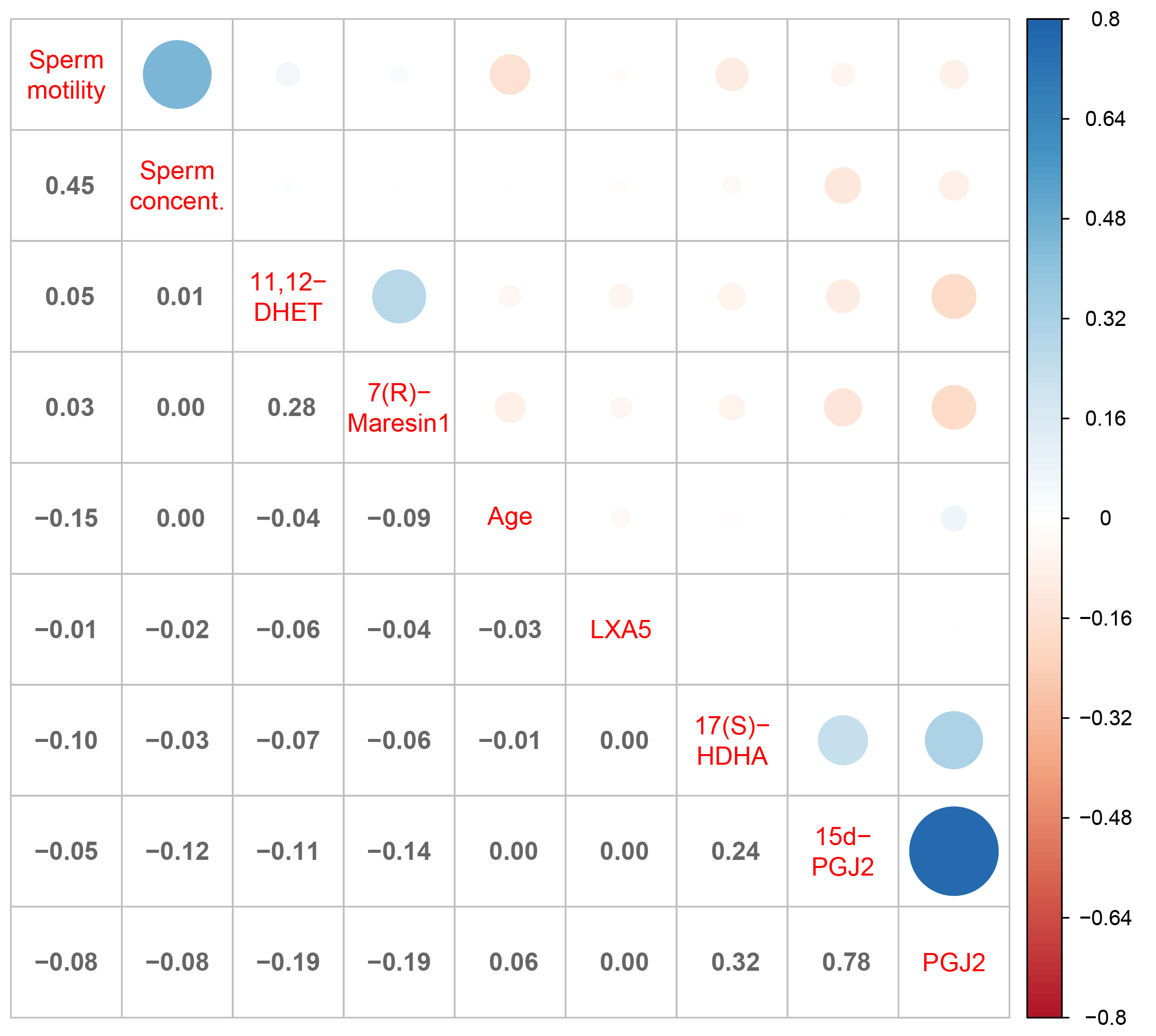

Supplement: Supplementary Figure 1 — Analysis of collinearity among different risk factors. [file Image_1.jpeg]
