# Supplementary material for: Relevance of PUFA-derived metabolites in seminal plasma to male infertility
Source: Front Endocrinol (Lausanne). 2023 May 22;14:1138984. doi: 10.3389/fendo.2023.1138984 (PMC10240070; doi:10.3389/fendo.2023.1138984)
Supplement: Supplementary Table 2 — Values of PUFA-derived metabolites determined by LC-MS in the seminal plasma of men with normozoospermia. [file Table_2.docx]

TableS2: Values of PUFA metabolites determined by LC–MS in seminal plasma

|  |  |  |  |  | FDR | | |
| --- | --- | --- | --- | --- | --- | --- | --- |
|  | Norm  Fertile | Norm  Infertile | OA  Fertile | OA  Infertile | Norm  Infertile VS Norm  fertile | OA  Infertile VS OA  fertile | OA  Infertile VS Norm  fertile |
| **N** | 267 | 109 | 121 | 67 | a: means FDR<0.05 | b:means FDR<0.05 | c:means FDR<0.05 |
| **LOX PATHWAY MATABOLITES** |  | | | | | | |
| 7(R)-Maresin 1 ^abc^ | 2.71 (2.76) | 1.12 (2.03) | 2.28 (2.74) | 0.85 (1.63) | 9.24E-07 | 0.00119 | 0.0000022 |
| 17(S)-HDHA^abc^ | 4.43 (1.22) | 4.99 (1.49) | 4.44 (1.28) | 5.09 (1.38) | 0.000903 | 0.00949 | 0.00089 |
| 9-HODE^ac^ | 1.76 (0.96) | 2.16 (1.21) | 1.90 (1.24) | 2.19 (1.10) | 0.00297 | 0.223 | 0.00689 |
| 13-HODE | 3.51 (1.09) | 3.40 (1.31) | 3.74 (1.16) | 3.57 (1.10) | 0.579 | 0.473 | 0.767 |
| 5-HETE^ac^ | 0.47 (0.43) | 0.66 (0.68) | 0.47 (0.35) | 0.62 (0.50) | 0.00377 | 0.0532 | 0.0408 |
| 12-HETE^c^ | 3.00 (1.43) | 3.19 (1.50) | 3.24 (1.50) | 3.51 (1.52) | 0.381 | 0.422 | 0.0343 |
| 15-HETE | 6.61 (1.55) | 6.47 (1.43) | 6.49 (1.35) | 6.17 (1.15) | 0.579 | 0.223 | 0.066 |
| LTB4 | 0.12 (0.21) | 0.12 (0.21) | 0.16 (0.28) | 0.15 (0.27) | 0.86 | 0.998 | 0.38 |
| LXA5^abc^ | 1.26 (1.19) | 2.31 (1.01) | 1.34 (1.29) | 2.45 (1.03) | 1.63E-13 | 9.94E-08 | 8.18E-12 |
| RvE1 | 5.88 (0.76) | 5.91 (0.71) | 5.99 (0.67) | 5.87 (0.76) | 0.82 | 0.433 | 0.955 |
| **P450 PATHWAY MATABOLITES** |  |  |  |  |  |  |  |
| 20-HETE | 0.61 (0.67) | 0.45 (0.49) | 0.61 (0.67) | 0.51 (0.46) | 0.0593 | 0.433 | 0.354 |
| 5(6)-EET | 0.43 (0.48) | 0.41 (0.33) | 0.34 (0.38) | 0.44 (0.31) | 0.86 | 0.188 | 0.933 |
| 8(9)-EET | 0.26 (0.40) | 0.24 (0.33) | 0.20 (0.32) | 0.20 (0.33) | 0.82 | 0.998 | 0.38 |
| 11(12)-EET | 0.46 (0.61) | 0.45 (0.54) | 0.40 (0.50) | 0.40 (0.38) | 0.86 | 0.998 | 0.477 |
| 14(15)-EET^a^ | 1.18 (1.65) | 1.69 (2.19) | 1.39 (2.00) | 1.49 (2.08) | 0.0384 | 0.842 | 0.35 |
| 5,6-DHET ^bc^ | 0.07 (0.12) | 0.04 (0.06) | 0.08 (0.14) | 0.03 (0.03) | 0.0936 | 0.0298 | 0.0343 |
| 8,9-DHET | 0.25 (0.28) | 0.27 (0.24) | 0.22 (0.24) | 0.18 (0.16) | 0.734 | 0.407 | 0.0943 |
| 11,12-DHET ^abc^ | 0.35 (0.30) | 0.23 (0.23) | 0.35 (0.29) | 0.24 (0.23) | 0.000903 | 0.0383 | 2.66E-02 |
| 14,15-DHET | 0.36 (0.31) | 0.41 (0.22) | 0.38 (0.30) | 0.40 (0.29) | 0.247 | 0.708 | 0.415 |
| **COX PATHWAY MATABOLITES** |  |  |  |  |  |  |  |
| PGD2^b^ | 1.79 (1.21) | 2.00 (1.25) | 1.64 (1.05) | 2.07 (1.16) | 0.247 | 0.0383 | 0.165 |
| PGE2 | 8.00 (1.17) | 8.26 (0.94) | 8.07 (1.00) | 8.15 (1.03) | 0.0744 | 0.708 | 0.415 |
| PGI2^a^ | 2.48 (2.07) | 1.78 (2.30) | 2.31 (2.13) | 1.91 (2.51) | 0.0122 | 0.422 | 0.106 |
| 15d- PGJ2^ac^ | 2.63 (0.92) | 3.07 (1.10) | 2.62 (0.89) | 2.96 (1.11) | 0.000603 | 0.0645 | 0.0378 |
| PGJ2^abc^ | 1.40 (1.94) | 3.52 (2.85) | 1.53 (2.02) | 3.73 (2.72) | 4.42E-14 | 5.20E-08 | 3.60E-13 |
| TXB2 | 1.54 (0.75) | 1.68 (0.89) | 1.60 (0.67) | 1.66 (0.86) | 0.202 | 0.708 | 0.38 |
| **Non-enzymatic**  **MATABOLITES** |  |  |  |  |  |  |  |
| 8-iso-PGF2α | 4.96 (1.54) | 4.99 (1.73) | 5.11 (1.53) | 4.98 (1.73) | 0.88 | 0.708 | 0.955 |

OA: Oligoasthenotspermia

Norm: Normozoospermia

Values are the mean ± S.D. and are expressed as μg/L（natural log transformation） seminal plasma.
