# Supplementary material for: Relevance of PUFA-derived metabolites in seminal plasma to male infertility
Source: Front Endocrinol (Lausanne). 2023 May 22;14:1138984. doi: 10.3389/fendo.2023.1138984 (PMC10240070; doi:10.3389/fendo.2023.1138984)
Supplement: Supplementary Table 3 — Values of PUFA-derived metabolites in propensity score-matched cohorts. [file Table_3.docx]

TableS3: Values of PUFA metabolites in propensity score−matched cohorts

|  |  |  |  |  | FDR | |
| --- | --- | --- | --- | --- | --- | --- |
|  | Norm  Fertile | Norm  Infertile | OA  Fertile | OA  Infertile | Norm  Infertile VS Norm fertile | OA  Infertile VS OA  fertile |
| **N** | 100 | 100 | 64 | 64 | a: means FDR<0.05 | b:means FDR<0.05 |
| **LOX PATHWAY MATABOLITES** |  | | | | | |
| 7(R)-Maresin 1 ^ab^ | 2.16 (2.59) | 1.14 (2.05) | 2.39 (2.81) | 0.87 (1.66) | 0.00205 | 0.00512 |
| 17(S)-HDHA^ab^ | 4.40 (1.32) | 5.00 (1.49) | 4.43 (1.34) | 5.17 (1.32) | 0.0273 | 0.00866 |
| 9-HODE^a^ | 1.66 (0.97) | 2.17 (1.22) | 1.89 (1.31) | 2.24 (1.10) | 0.0181 | 0.214 |
| 13-HODE | 3.45 (1.07) | 3.42 (1.33) | 3.81 (1.18) | 3.61 (1.05) | 0.791 | 0.551 |
| 5-HETE | 0.52 (0.53) | 0.68 (0.69) | 0.49 (0.35) | 0.63 (0.50) | 0.106 | 0.192 |
| 12-HETE | 2.91 (1.41) | 3.21 (1.54) | 3.16 (1.43) | 3.48 (1.51) | 0.208 | 0.357 |
| 15-HETE | 6.39 (1.60) | 6.53 (1.42) | 6.56 (1.28) | 6.19 (1.16) | 0.943 | 0.306 |
| LTB4 | 0.14 (0.22) | 0.10 (0.18) | 0.16 (0.30) | 0.13 (0.20) | 0.286 | 0.603 |
| LXA5^ab^ | 1.43 (1.45) | 2.33 (1.02) | 1.42 (1.44) | 2.45 (1.05) | 0.00000298 | 0.000102 |
| RvE1 | 5.92 (0.57) | 5.94 (0.72) | 6.02 (0.53) | 5.88 (0.77) | 0.943 | 0.406 |
| **P450 PATHWAY MATABOLITES** |  |  |  |  |  |  |
| 20-HETE^a^ | 0.59 (0.61) | 0.42 (0.46) | 0.64 (0.70) | 0.51 (0.47) | 0.0414 | 0.357 |
| 5(6)-EET | 0.44 (0.51) | 0.42 (0.33) | 0.38 (0.41) | 0.45 (0.30) | 0.597 | 0.406 |
| 8(9)-EET | 0.26 (0.42) | 0.25 (0.34) | 0.17 (0.25) | 0.20 (0.33) | 0.741 | 0.811 |
| 11(12)-EET | 0.40 (0.49) | 0.46 (0.55) | 0.44 (0.53) | 0.39 (0.37) | 0.624 | 0.673 |
| 14(15)-EET^a^ | 1.14 (1.62) | 1.62 (2.10) | 1.47 (1.96) | 1.29 (1.90) | 0.0488 | 0.698 |
| 5,6-DHET ^b^ | 0.06 (0.11) | 0.04 (0.06) | 0.06 (0.09) | 0.03 (0.03) | 0.253 | 0.0283 |
| 8,9-DHET | 0.23 (0.27) | 0.27 (0.25) | 0.20 (0.19) | 0.17 (0.16) | 0.522 | 0.457 |
| 11,12-DHET ^ab^ | 0.32 (0.27) | 0.22 (0.22) | 0.33 (0.24) | 0.22 (0.21) | 0.0181 | 0.0283 |
| 14,15-DHET | 0.38 (0.26) | 0.42 (0.22) | 0.37 (0.28) | 0.39 (0.28) | 0.314 | 0.698 |
| **COX PATHWAY MATABOLITES** |  |  |  |  |  |  |
| PGD2^b^ | 1.78 (1.02) | 2.04 (1.27) | 1.60 (1.09) | 2.17 (1.10) | 0.255 | 0.0208 |
| PGE2 | 8.03 (0.94) | 8.25 (0.95) | 8.04 (0.95) | 8.18 (1.02) | 0.208 | 0.551 |
| PGI2 | 2.19 (2.19) | 1.64 (2.26) | 2.30 (2.08) | 1.72 (2.40) | 0.2 | 0.357 |
| 15d- PGJ2^a^ | 2.71 (0.84) | 3.12 (1.10) | 2.62 (0.87) | 2.95 (1.10) | 0.0224 | 0.181 |
| PGJ2^ab^ | 1.71 (2.21) | 3.67 (2.84) | 1.47 (2.00) | 3.90 (2.67) | 2.98E-06 | 1.26E-06 |
| TXB2 | 1.62 (0.73) | 1.69 (0.89) | 1.56 (0.67) | 1.65 (0.85) | 0.65 | 0.603 |
| **Non-enzymatic**  **MATABOLITES** |  |  |  |  |  |  |
| 8-iso-PGF2α | 5.18 (1.38) | 5.12 (1.66) | 5.02 (1.46) | 5.06 (1.70) | 0.731 | 0.957 |

OA: Oligoasthenotspermia

Norm: Normozoospermia

Values are the mean ± (S.D.) and are expressed as μg/L（Log2 transformation） seminal plasma.
